# Type I interferon inhibits varicella-zoster virus replication by interfering with the dynamic interaction between mediator and IE62 within replication compartments

**DOI:** 10.1186/s13578-016-0086-6

**Published:** 2016-03-16

**Authors:** Chia-Chi Ku, Yi-Hsuan Chang, Yun Chien, Tsung-Lin Lee

**Affiliations:** Institute of Immunology, College of Medicine, National Taiwan University, No.1 Section 1 Jen-Ai Road, Taipei, Taiwan

**Keywords:** Varicella-zoster virus, IE62, Type I interferons, Mediator complex, Replication compartment

## Abstract

**Background:**

Varicella-zoster virus (VZV) is the causative agent of varicella and zoster. The immediate-early protein, IE62 is the predominant VZ virion tegument protein, transactivating the expression of all kinetic classes of VZV genes. IE62 is localized to punctae that form DNA replication compartments in the nuclei of VZV infected cells. The morphological changes and the increase in the size of replication compartments that express IE62 are correlated with production of VZ virions. Mammalian Mediator serves as a coactivator of IE62 and functions by bridging DNA-binding transcription factors¸ RNA polymerase II (RNAP II) and their target DNAs for VZV replication. While VZV is highly sensitive to type I interferons (IFNs), how IFN-α inhibits early events during VZV replication is poorly understood.

**Results:**

In this study, we performed in situ analysis to investigate the effects of IFN-α on the dynamic interactions of IE62 with the Mediator MED25 subunit and the RNAP II negative regulator cycle-dependent kinase 8 (CDK8) in VZV infected cells by confocal immunofluorescence. We found that in addition to dose-dependent inhibition of the yields of infectious virus by IFN treatment, IFN-α prominently impeded the development of large IE62^+^ nuclear compartments and significantly decreased transcription of VZV genes. Both the expression level and stable recruitment of MED25 to IE62^+^ replication compartments were inhibited by IFN-α. While IFN-α treatment upregulated CDK8 expression, redistribution and recruitment of CDK8 to IE62^+^ replication compartments in infected cells was not affected by VZV.

**Conclusion:**

IFN-α exerts multiple inhibitory activities against virus infections. In this study, we provide visionary demonstration that continuous translocation of MED25 into VZV replication compartments ensures production of virions. IFN-α greatly impedes the formation of a stable complex between IE62 and the Mediator complex thereby suppresses VZV gene transcription. Our demonstration that IFN-α-induced antiviral effect against VZV infection is through inhibiting the reorganization of nuclear components uncovers a novel function of IFN-α. Targeting the interaction between IE62 and MED25 may offer a novel approach to the development of antiviral agents against VZV infection.

## Background

Varicella-zoster virus (VZV) is an alphaherpesvirus. It has a double-stranded DNA genome of about 125,000 base pairs which encodes for at least 70 unique open reading frames (ORFs). Primary VZV infection causes varicella (chickenpox) characterized by viremia and skin lesions. VZV can be reactivated in sensory ganglia from latency to cause zoster (shingles) [1].

Type I interferons (IFNs) including IFN-α and IFN-β constitute a potent innate defense system against virus infections [2]. IFN-α treatment dose-dependently inhibits the production of VZV in human foreskin fibroblast cells [3] and the expression of VZV immediate-early, early and late proteins are suppressed in IFN-α treated cells [4]. We have previously demonstrated that IFN-α is induced in uninfected epidermal keratinocytes that surround VZV lesions. Blocking IFN-α/β receptor-mediated signaling enhances VZV replication and promotes viral spread in human skin xenografts in severe combined immunodeficiency (SCID) mice [5]. While IFN-induced activation of antiviral protein kinase R correlates with inhibition of VZV replication [6], how IFN-α inhibits early events of VZV replication is poorly understood.

VZV immediate-early (IE) 62 acts to induce the expression of other immediate early (IE), early (E), and late (L) VZV genes before any other viral proteins are synthesized [1]. It is reported that Mediator, a multi-subunit coactivator of RNA polymerase II (RNAP II) serves as co-activator for VZV IE62 to transactivate genes during lytic VZV infection. Mediator is a global transcription regulator conserved in yeast and mammalian cells [7]. Physical interaction of VZV IE62 with the Mediator 25 (MED25) subunit is required for IE62 transactivation activity [8].

Single cell analysis of VZV-infected cells showed that VZV DNA initially accumulates in small punctae near the nuclear rim. The DNA punctae increase in size as viral DNA replicates and acquires a globular pattern over time. IE62 is found to localize in large DNA punctae as well as the globular structures [9]. The facts that IE62 recruits cellular transcription factors and RNAP II to the TATA sites within the VZV promoters [10] and that MED25 enhances IE62 transactivation activity led us to investigate whether IFN-α might interfere with VZV replication through inhibiting MED25 recruitment to IE62-expressing replication compartments.

While MED25 positively activates gene transcription, the 4-subunit CDK8 module (consisting of cell cycle-dependent kinase 8 (CDK8), cyclin C, MED12 and MED13) sterically blocks the interaction of Mediator with RNAP II and regulates RNAP II-dependent transcription initiation [11, 12]. It was recently reported that type I and type II IFNs induce nuclear expression of CDK8 [13]. Since VZV gene expression is RNAP-II dependent and IE62 inhibits TANK-binding kinase (TBK-1) activity, which results in the blockage of IFN-β production [14], it is of interest to know whether IFN-α treatment induces CDK8 expression and whether VZV infection suppresses IFN-α-induced CDK8 expression and distribution.

The highly cell-associated nature of VZV replication is a primary obstacle to studies of the early events during VZV replication in newly infected cells. Since only low titers of cell-free VZV can be recovered from infected cells in culture, synchronous infection is not possible. Using infected cells as inoculum results in the presence of both inoculum cells and newly infected cells which are indistinguishable by using viral protein expression as markers. However, fluorescent dye labeled inoculum cells coupled with confocal immunofluorescence microscopic analysis made it possible to document the spatiotemporal expression of major VZV proteins [9]. The advancement in microscopy technology by digitalizing fluorescence intensity allows comparisons of protein expression in subcellular localizations in tissue sections as well as cultured cells [15–17]. Taking advantage of these technologies, we did in situ analysis to investigate the effects of IFN-α on the dynamics of MED25 and CDK8 distribution in IE62-expressing replication compartments. We found that stable recruitment of MED25 to VZV replication compartments positively correlated with the progressive development of IE62-expressing large and large globular punctae in VZV-infected cells. VZV gene transcription, the size of IE62-expressing nuclear compartments and recruitment of MED25 to the replication compartments were all reduced in IFN-α treated cells. Interestingly, IFN-α treatment upregulated CDK8 expression although CDK8 redistribution and recruitment to IE62^+^ replication compartments in VZV infected cells was not affected. Our findings reveal that IFN-α treatment interferes with intranuclear redistribution of Mediator and blocks VZV gene transcription.

## Results

### IFN-α treatment inhibits IE62 expression in VZV-infected HELF cells

**Fig. 1:**
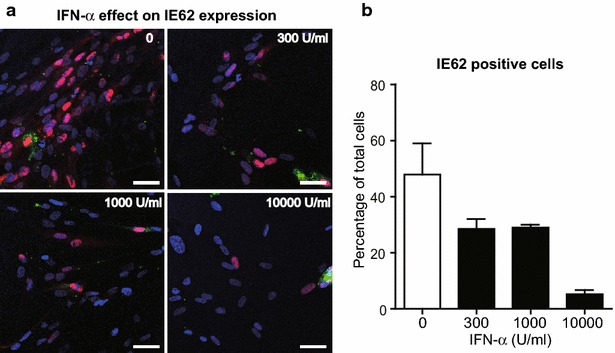
Dose dependent effects of IFN-α on IE62 expression. Primary human embryonic fibroblasts (HELFs) were untreated or treated with IFN-α at 300, 1000 or 10,000 U/ml for 24 h followed by inoculation of green fluorescent dye CFSE-labeled VZV infected cells. **a** The cell monolayer was fixed at 24 h, stained with primary antibody to VZV IE62 and detected with donkey anti-rabbit IgG (*red*). Green stained cells were inoculated VZV-infected cells. Cells were counterstained with DAPI and analyzed by confocal microscopy. Shown are representative illustrations from at least three independent experiments. *Scale bar* 100 μm. **b** IE62 positive cells were counted for each group of cells in the total of 20–200 cells in the same coverslip. The percentages of cells that expressed IE62 were thus calculated. Shown is the average from two independent experiments with *error bars*

Primary human embryonic lung fibroblast (HELF) monolayers were pretreated with different concentrations of IFN-α before addition of green fluorescent dye CFSE-labeled VZV-infected cells. Confocal microscopic analysis showed that CFSE^+^ VZV inoculum cells were surrounded by newly infected cells expressing IE62 (Fig. 1a). IFN-α treatment reduced nuclear IE62 expression in the VZV-infected monolayers in a dose dependent manner (Fig. 1a). Typical large VZV plaques with syncytia formation were not observed in IFN-α treated monolayers. The percentage of IE62^+^ cells was 59 % without treatment and was reduced to 32, 28 and 6.7 % with treatment of 300, 1000, 10,000 U/ml of IFN-α, respectively (Fig. 1b). High dose of IFN-α significantly inhibited the initiation of infection by IE62 expression in VZV infected cells.

### IFN-α treatment effectively inhibits IE62-dependent expression of VZV genes of all classes and reduces the production of infectious virions

**Fig. 2:**
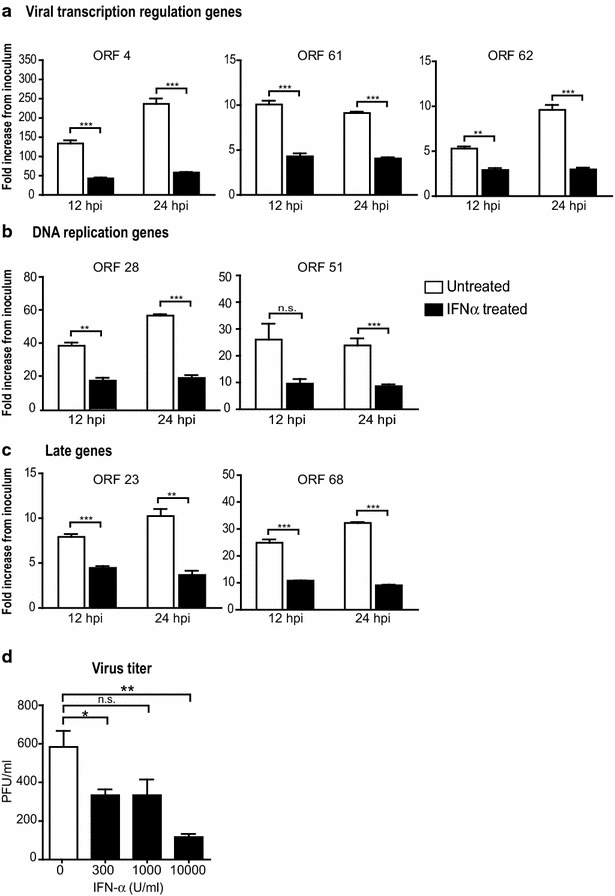
IFN-α inhibits VZV gene expression. Expression of selected VZV genes representing immediate-early (**a**), early (**b**) and late (**c**) genes in untreated or IFN-α treated HELFs at 12 and 24 h after incubation with VZV infected cells was measured by quantitative real-time PCR. Expression of each gene at 12 and 24 h was normalized to the expression of *Gapdh*. The levels of expression were calculated by fold increase compared to the corresponding gene expressed in cells at 0 h in which cells were harvested immediately after inoculation with VZV infected cells. Each *bar* represents the mean ± SEM of triplicate measurements from one representative of two independent experiments. The difference in expression of each gene expressed in untreated and IFN-α treated cells at 12 or 24 h was compared by Student’s t test. **d** Virus yields from untreated cells or cells treated with varying concentrations of IFN-α were evaluated by infectious focus assay at 24 h post infection. Each *bar* represents the mean ± SEM of triplicate titrations from three independent experiments (unpaired Student’s t test). (**p < 0.01; ***p < 0.001; not significant (n.s.), p > 0.05)

To quantify the effect of IFN-α on VZV gene transcription, we analyzed seven VZV genes that are otherwise normally expressed during immediate early (IE) (ORF4, ORF61 and ORF62), early (E) (ORF28 and ORF51) and late (L) (ORF23, ORF68) phases of infection [1, 4]. Quantitative RT-PCR results showed that while ORFs 4, 61, 62, 28, 51, 23, and 68 were abundantly expressed at 12 and 24 h after VZV infection, IFN-α treatment reduced the levels of VZV IE (ORFs 4, 61 and 62) transcripts by two–threefold at 12 h, and remained low at 24 h after treatment (*p* < 0.001) (Fig. 2a). Early and late genes transcripts including ORFs 28, 51, 23, and 68 were also reduced significantly in IFN-α treated cells compared to untreated VZV-infected HELF over the 24 h time course (*p* < 0.001) (Fig. 2b, c). These experiments demonstrated that IFN-α effectively inhibits IE gene transcription and the subsequent expression of VZV genes of all classes. Viral yields from IFN-α treated cells were also reduced in a dose dependent manner compared to untreated cells (Fig. 2d). These results showed that IFN-α treatment inhibits IE62-dependent VZV gene expression and reduced the production of progeny virions.

### IFN-α blocks the progressive development of IE62-expressing replication compartments from small and large to large gobular patterns

**Fig. 3:**
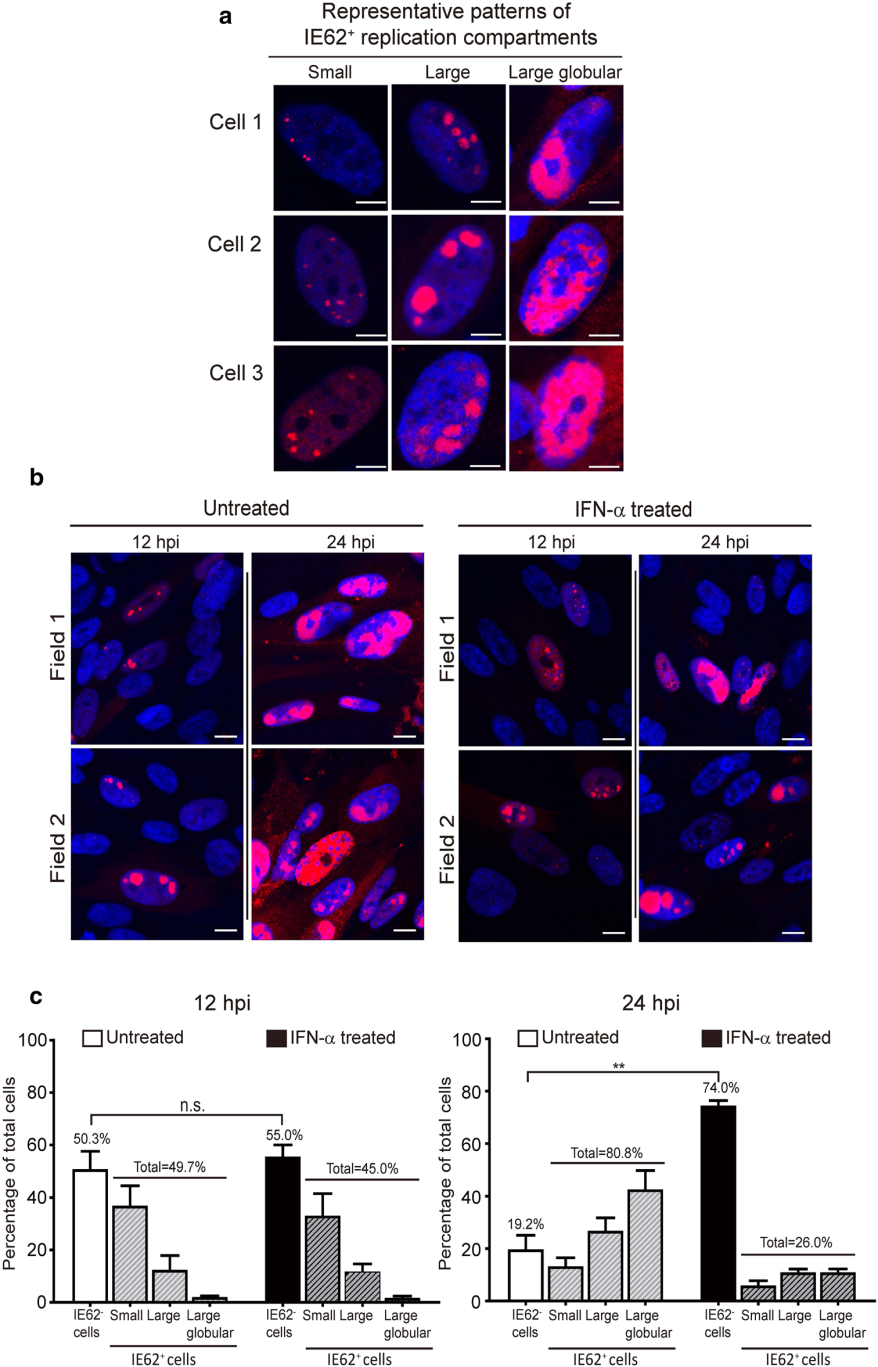
IFN-α affects the size and morphology of IE62-expressing replication compartments. **a** Three representative cells from confocal microscopy images illustrate small punctate (<3.2 μm^2^), large punctate (3.2–16 μm^2^) or large globular pattern (>16 μm^2^) based on IE62 staining. *Scale bar* 5 μm. **b** Untreated or IFN-α treated HELF cell monolayers at 12 and 24 h post VZV infection were stained with anti-IE62 antibody. Shown are two representative fields from microscopic confocal images and illustrate that IFN-α treatment inhibits the progression of large globular replication compartments compared with untreated cell monolayer. *Scale bar* 10 μm. **c** Confocal microscopic images from 20 fields of IE62 stained cell monolayers from two independent experiments were analyzed for the size and morphology of IE62-expressing replication compartments. 30–90 untreated and IFN-α treated cells at 12 or 24 h post VZV inoculation were analyzed. Each *bar* represents the mean percentage of total cells, which were uninfected, or IE62 positive cells expressing small, large or large globular morphology. Differences in IE62 expression between untreated and IFN-α treated cells were tested by unpaired Student’s t test

Nuclear IE62 is known to translocate and accumulate in the cytoplasm in VZV-infected cells at late stage of infection [18]. We analyzed IE62 expression patterns while IE62 still remained in the nuclei of VZV-infected cells. IE62 appears at nuclear membranes early in VZV replication and IE62-expressing nuclear domains increase in size and change in morphology as viral DNA transcription proceeds [9]. We utilized the changing morphology of IE62 nuclear expression patterns as an indicator to study the effect of IFN-α on VZV replication compartments at the single cell level. In VZV-infected cells, IE62 accumulated initially in distinct small (<3.2 μm^2^/area) and large (3.2–16 μm^2^/area) punctae forms and changed to large globular pattern (>16 μm^2^/area) as infection progressed (Fig. 3a). There was no difference in the distribution of the small, large punctae and large globular pattern of IE62 expression between IFN-α-treated and untreated cells at 12 h (Fig. 3b, c). However, IFN-α treatment significantly inhibited the progression of IE62-expressing foci from small to large punctae and the large globular pattern by 24 h, and there was a high percentage of uninfected cells in the monolayer (Fig. 3b). Importantly, while the percentage of IFN-α-treated cells (45 ± 5.01 %) that expressed IE62 was comparable to untreated cells (49.75 ± 7.3 %) at 12 h, it was significantly lower (26.0 ± 1.4 %) than untreated cells (80.8 ± 5.9 %) at 24 h (*P* < 0.01) (Fig. 3c). These experiments show that IFN-α pretreatment had significant impact on the development of VZV replication compartments but not on the initiation of IE62 expression.

### IFN-α treatment blocks the stable recruitment of MED25 into IE62-expressing replication compartments

**Fig. 4:**
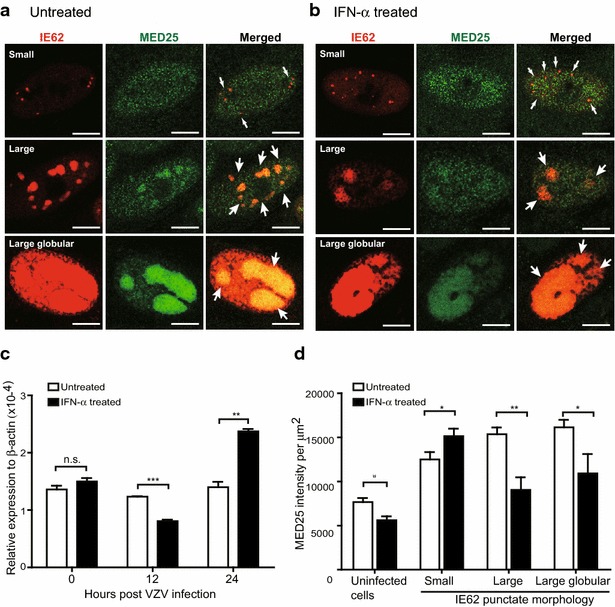
IFN-α reduces nuclear MED25 expression and interferes with its redistribution to IE62-expressing replication compartments. Untreated or IFN-α treated HELF monolayers were fixed in 4 % PFA at 12 or 24 h post VZV inoculation. The cell monolayers were double stained with anti-MED25 (*green*) and anti-IE62 (*red*) antibodies for confocal microscopic analysis. Representative double immunofluorescence stained single cells from untreated (**a**) or IFN-α treated (**b**) illustrated MED25 expression in IE62-expressing replication compartments in small, large or large globular pattern. *Arrows* shown in merged images indicate colocalization of IE62 and MED25. **c** Expression of *Med25* mRNA in untreated or IFN-α-treated HELFs at 12 and 24 h after incubation with VZV-infected or mock-infected cells was measured by qRT-PCR. The level of expression was normalized to the expression of human *β*-*actin* and expressed as relative expression to human *β*-*actin.* Each *bar* represents the mean ± SEM of triplicate measurements from one experiment. **d** MED25 integrated intensity from each cell was measured by MetaMorph software. The averages of MED25 integrated intensity in untreated (*white bars*) or IFN-α treated cells (*black bars*) without VZV inoculation or with VZV infection are shown in (**d**). MED25 integrated intensity expressed in the nucleus of uninfected HELF cells was measured based on DAPI stain. The averages of MED25 integrated intensity for IE62-expressing cells in various morphological forms in untreated or IFN-α treated cells are shown. Each *bar* represents the mean ± SEM of MED25 integrated intensity from 14–16 cells (untreated group) or 20–30 cells (IFN-α treated group) analyzed. Differences between each groups were tested by unpaired Student’s t test (**p < 0.01; ***p < 0.001; not significant (n.s.), p > 0.05). *Scale bar* 5 μm

Nuclear protein MED23 and MED25 are subunits of the mammalian Mediator complex known to be associated with the IE62-mediated transcriptional complex [19]. Confocal immunofluorescence analysis showed that MED23 which is typically diffuse in nuclei redistributes during the early phase of VZV infection to a pattern that overlapped completely with IE62. MED23 returns to a diffuse nuclear distribution in the late phase when IE62 is translocated to the cytoplasm [20]. To investigate the effect of IFN-α on the dynamic interaction between IE62 and the Mediator subunit, we studied the redistribution of MED25 to IE62 compartments and quantified its expression level in untreated and IFN-α treated cells. Similar to MED23, VZV infection induced MED25 redistribution and translocation to IE62-expressing nuclear domains (Fig. 4a, b). While MED25 protein colocalized with IE62 punctae in small, large and large globular morphological forms, MED25 was more prominent in large punctae and large globular foci (Fig. 4a). Interestingly, while MED25 redistribution was also observed in the nuclei of IFN-α treated IE62-expressing cells, the level of MED25 that co-localized with IE62 was much reduced (Fig. 4b). *Med25* mRNA was stably expressed in untreated and VZV-infected cells over the first 24 h after infection. While IFN-α treatment did not affect *Med25* mRNA transcription in uninfected cells, it significantly reduced *Med25* expression at 12 h and increased its expression at 24 h after VZV infection (Fig. 4c). Single cell analysis shows that MED25 expression was reduced in IFN-α-treated cells without VZV infection (Fig. 4d). The level of MED25 in IE62^+^ compartments was increased and its intensity increased as the size of IE62^+^ foci increased (Fig. 4d). With IFN-α treatment, while there was an increase of MED25 in small IE62^+^ punctae, the intensity of MED25 in the large globular IE62^+^ foci was significantly lower than in untreated cells (Fig. 4d). These results indicate that VZV replication is associated with translocation of MED25 and its stable and continuous presence in IE62^+^ replication compartments. IFN-α is likely to interfere with VZV replication by inhibiting its co-localization with IE62 in replication compartments rather than inhibiting transcription of *Med25* gene, thus impairing the progression of VZV infection.

### IFN-α increases nuclear CDK8 expression and its association with IE62-expressing replication compartments

**Fig. 5:**
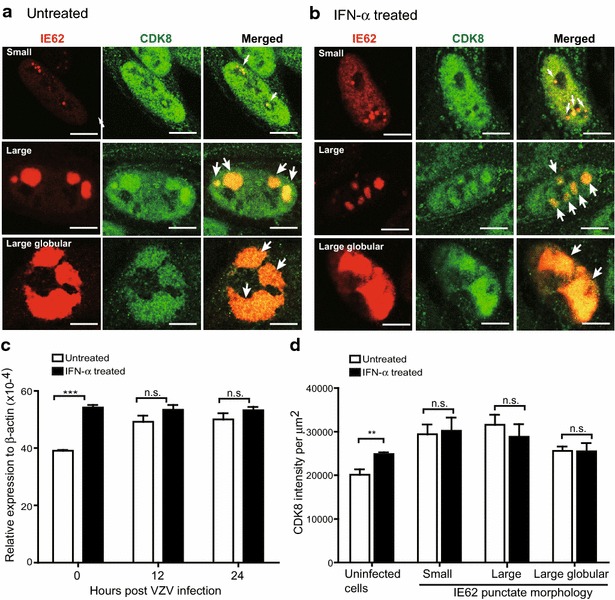
IFN-α increases nuclear CDK8 expression and its association with IE62-expressing replication compartments. Untreated or IFN-α treated HELF monolayers were fixed in 4 % PFA at 12 or 24 h post VZV inoculation. The cell monolayers were double stained with anti-CDK8 (*green*) and anti-IE62 (*red*) antibodies for confocal microscopy analysis. Representative double immunofluorescence stained single cells from untreated (**a**) or IFN-α treated (**b**) illustrated CDK8 expression in IE62-expressing replication compartments in small, large or large globular pattern. *Arrows* shown in merged photographs indicate colocalization of IE62 and CDK8. **c** Expression of *Cdk8* mRNA in untreated or IFN-α treated HELFs at 12 and 24 h after incubation with VZV-infected or mock-infected cells was measured by qRT-PCR. The level of expression was normalized to the expression of human *β*-*actin* and expressed as relative expression to human *β*-*actin.* Each bar represents the mean ± SEM of triplicate measurements from one experiment (unpaired Student’s t test). **d** CDK8 integrated intensity from each cell was measured by MetaMorph software. The averages of CDK8 integrated intensity in untreated (*white bars*) or IFN-α treated cells (*black bars*) without VZV inoculation or with VZV infection are shown in (**d**). CDK8 integrated intensity expressed in the nucleus of uninfected HELF cells was measured based on DAPI stain. The averages of CDK8 integrated intensity for IE62^+^ cells in various morphological forms in untreated or IFN-α treated cells are shown. Each *bar* represents the mean ± SEM of CDK8 integrated intensity from 20–40 cells (untreated group) or 20–55 cells (IFN-α treated group) analyzed. Differences between each group were tested by unpaired Student’s t test (**p < 0.01; ***p < 0.001; not significant (n.s.), p > 0.05). *Scale bar* 5 μm

We studied the interaction between CDK8 with IE62 and the effects of IFN-α treatment in VZV-infected cells. Confocal microscopic analysis shows that CDK8 was redistributed and recruited to IE62^+^ replication compartments upon infection whether or not cells were pretreated with IFN-α (Fig. 5a). There was no difference in CDK8 distribution and association with IE62 in infected cells with or without IFN-α treatment (Fig. 5b). Expression of *Cdk8* mRNA was significantly upregulated by IFN-α treatment and remained stable after VZV infection (Fig. 5c). Single cell analysis shows that CDK8 expression was enhanced in IFN-α treated cells before VZV infection (Fig. 5d). It is interesting to note that CDK8 was also recruited to IE62 compartments in infected but untreated cells (Fig. 5d). Our data demonstrate that while CDK8 expression is induced by IFN-α treatment and translocation of CDK8 into IE62^+^ replication compartments in VZV-infected cells occurs regardless of IFN-α treatment, further investigation is required to define the role for CDK8 in VZV infection.

## Discussion

In this study, we analyzed expression and intranuclear localization of the IE62 major transactivating protein of VZV and the cell proteins, MED25 and CDK8 at a single cell level in order to better understand how IFN-α impairs VZV replication. IFN-α treatment impaired the progression of IE62-expressing compartments from small to large globular punctae in VZV-infected cells, which was associated with a reduction of transcription of all kinetic classes of VZV genes. While MED25 was dramatically re-localized to VZV replication compartments in VZV-infected cells, IFN-α treatment interfered with this process. Interestingly, CDK8 was redistributed and recruited to IE62^+^ replication compartments upon VZV infection whether or not cells were pretreated with IFN-α and IFN-α treatment upregulated CDK8 expression overall. This study indicates that IFN-α inhibits VZV gene expression, virus spread in monolayers and production of progeny virus particles by interfering with the intranuclear redistribution of Mediator to IE62-expressing replication compartments.

We have previously reported that IFN-α induction inhibits VZV spread and formation of lesions in human skin xenografts in SCID mice in vivo [5]. We also observed that VZV proteins were expressed in epidermal cells within VZV lesions but not in the neighboring keratinocytes. The in situ single cell analysis data presented here showed that while IFN-α treatment did not affect the initiation of IE62 expression, it suppressed the development of globular VZV replication compartments. It is our speculation that inhibition of the maturation of replication compartment that is associated with productive infection is likely the mechanism of how IFN-α causes restricted viral gene transcription.

The cell Mediator complex governs eukaryotic gene transcription through extensive interactions with RNAP II and both general and gene-specific transcription factors [21]. The pull-down assays showed that MED25 is the cell substrate targeted by the IE62 activation domain [8]. Silencing MED25 by small RNA interfered with the interaction between MED25 and IE62 and inhibited the IE62-mediated promoter activity [8]. Our results demonstrated that stable recruitment of MED25 to IE62-expressing compartments ensures productive VZV replication. With regard to IFN-α suppression of VZV, we found that transcription of *Med25* is decreased in IFN-α treated cells after VZV infection despite that the antiviral response does not block the translocation of MED25 to small IE62 punctae. However, IFN-α significantly hinders continuous recruitment of MED25 to IE62-expressing replication compartments. Thus, IFN-α has the capacity to inhibit VZV infection by impairing the formation of replication compartments and blocking MED25 translocation to these compartments where viral gene transcription occurs in newly infected cells.

Reorganization of viral and cellular macromolecules within the nucleus of infected cells to form replication compartments provides a platform which ensures efficient viral replication for DNA viruses [22]. Like in other herpesviruses, transcription of VZV genome during lytic infection requires binding of RNAP II and other cellular transcription factors to IE promoters [10]. Although pull-down assays showed that the CDK8 module is excluded from IE62-interacting Mediator complex [8], we observed that VZV induces transcription of *Cdk8* mRNA and CDK8 is redistributed and translocated to IE62-expressing replication compartments in VZV-infected cells. These results suggest that CDK8 is involved in VZV replication. CDK8-dependent phosphorylation of host transcription factors is known to repress their transactivation activity [23, 24]. CDK8 does not directly bind to IE62. We observed that VZV infection triggers the redistribution of CDK8 to replication compartments even though it does not bind to IE62. The presence of CDK8 in replication compartments might play a role in shifting transcription from cellular genes to viral genes. Bancerk et al. showed that nuclear expression of CDK8 is important to both type I and type II IFN responses [13]. Our data show that IFN-α treatment reduced MED25 expression but increased that of CDK8. CDK8 nuclear redistribution occurred to VZV-infected cells with or without IFN-α treatment. Since MED25 and CDK8 reversibly bind to RNAP II [25], it is possible that reduced MED25 expression after IFN-α treatment may be important in keeping CDK8 in a functional state to inhibit viral replication. Further investigation of the role of CDK8 in VZV infection and in the IFN-α-mediated antiviral response is warranted.

The function of Mediator complex in the VZV replication has been demonstrated mainly by biochemical experiments [8, 19]. The molecular components of VZV replication compartments have been less well defined. The demonstration of temporal increase in the size of VZV DNA domains overlapping with IE62 expression [9] has prompted us to investigate the interactions of major viral transactivator IE62 and the Mediator complex within the VZV replication compartments. In this study, we provide visionary demonstration that continuous translocation of MED25 is essential for the maturation of VZV replication compartments. IFN-α treatment impedes the development of replication compartment partly through blocking the formation of a stable complex between IE62 and the Mediator complex thereby impairing viral replication. Our demonstration that IFN-α-induced antiviral effect against VZV infection is through inhibiting the reorganization of nuclear components uncovers a novel function of IFN-α. Targeting the interaction between IE62 and MED25 could represent a novel strategy to identify antiviral drugs for treating VZV infections.

## Conclusions

**Fig. 6:**
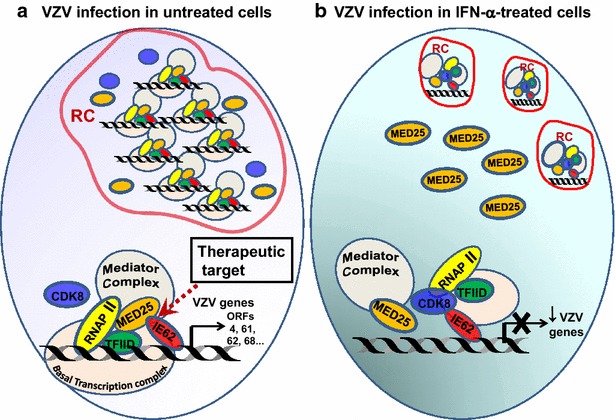
Speculative mechanism on how IFN-α inhibits VZV via interference of Mediator and IE62 within replication compartments. **a** During VZV infection, the major transactivator IE62 interacts with RNAP II, cellular transcription factor (*TF*) IID (TFIID), and the basal transcription complex which together form the replication compartment (*RC*) for viral DNA replication. Efficient VZV gene transcription requires recruitment of Mediator complex through IE62 and MED25 interaction which stabilizes RNAP II activity. Continuous translocation of MED25 into VZV RCs is essential for RC maturation (large globular pattern) and virion production. **b** IFN-α treatment impedes the formation of a stable complex between IE62 and Mediator complex, thus blocks RC maturation (small and large punctae). IFN-α induces the expression of CDK8 whose presence in RC may further inhibit the interaction between MED25 and IE62. Targeting the interaction between IE62 and MED25 (indicated by *dashed arrow* in **a**) may offer a novel approach to the development of antiviral agents against VZV infection

The antiviral effects of type I IFN has been extensively studied since it was first described by Isaacs and Lindenmann more than 50 years ago [26]. Type I IFN induces over 300 interferon-stimulated genes in treated cells and most of which interfere virus replication at the transcription and translation levels [27]. However, studies of the impacts of IFN-induced antiviral state on virus replication in situ are limited. With the advancement in digital microscopy technology, we investigated the dynamic interaction between viral transcription factor IE62 and Mediator within viral replication compartments at single cell level. Formation of a stable complex between IE62 and MED25 positively correlates with the maturation of replication compartments and production of VZV. IFN-α treatment significantly inhibits the development of mature replication compartments and impedes subsequent VZV gene transcription (as summarized in Fig. 6). Our study uncovers a novel function of IFN-α. We demonstrate that IFN-α-induced antiviral effect against VZV infection is through inhibiting the reorganization of nuclear components. Results from this study suggest that disruption of viral replication compartment by targeting the interaction between MED25 and IE62 has therapeutic potential for treating VZV infection.

## Methods

### Viruses, viral propagation and IFN-α treatment

Primary human embryonic lung fibroblasts (HELFs) were maintained in Minimal Essential Medium plus 10 % fetal calf serum and antibiotics. A clinical VZV isolate, strain S [28] was propagated in HELFs used at a maximal passage of 15–17 as previously described [29]. The virus stocks were stored in freezing medium with 10 % DMSO at −70 °C.

VZV-infected HELF were used to infect HELF monolayers at ratios of 1:1 or 1:10 infected to uninfected cells. The HELF monolayers were either left untreated or pretreated with recombinant human IFN alpha 2 (IFN-α) (Roferon^®^-A, Roche Diagnostics, IN) at 10,000 U/ml for 24 h before infection. Viral titers were assessed as previously described [5].

### Immunofluorescence and confocal microscopy

Methods using fluorescent dye to label the viral inoculum cells to differentiate the VZV infected inoculum (input cells) and the newly infected cells were modified from the previous study [9]. Briefly, 5 μM of the green membrane permeant fluorescent dye 5(6)-carboxyfluorescein diacetate N-succinimidyl ester (CFSE, Sigma-Aldrich) was added to a heavily infected HELF monolayer and incubated at 37 °C for 15 min. The succinimidyl side chain of CFSE is coupled to the amino groups of intracellular proteins via a very stable amide bond and achieves stable labeling of the cells because of the long-lived conjugates [30]. Labeled cells were washed in PBS, dispersed with trypsin, resuspended in PBS and used as inoculum cells. For infection, HELF were seeded onto sterile glass coverslips and incubated with labeled inoculum cells at a multiplicity of infection of one infected cell per 10 uninfected cells. Coverslips were incubated on ice for 30 min to allow the input cells to settle on the monolayer and then transferred to 37 °C to initiate infection. The primary antibodies used for immunofluorescence staining included mouse monoclonal antibodies against IE62 (H6), CDK8 (Bethyl Laboratories, Montgomery, TX) and MED25 (Thermo Scientific, Rockford, IL). For immunofluorescent staining, cells were fixed in 4 % paraformaldehyde (PFA) at 24 h, permeabilized with 2 % PFA + 0.2 % Triton X-100 at RT for 30 min, washed, and stained with primary antibodies at RT for 1 h. Primary antibodies were diluted in PBS with 1 % fish gelatin as follows: anti-IE62 (H6), 1:250; anti-CDK8, 1:500; anti-MED25, 1:50. After washing, cells were stained with AlexaFluo 488 (*green*) or AlexaFluo 555 (red)-conjugated secondary antibody (Life Technologies, Calsbad, CA). Fluorescence signals and the images were visualized and captured with a Leica SP5 confocal microscope system using HCX PL APO CS 100× NA 1.4 oil immersion planapochromative objectives with zoom 2× at Imaging Core facility, Genomic Research Center, Academia Sinica.

### Digital image analysis

The fluorescence intensity from the region of interest (ROI) of confocal images was measured using MetaMorph software. Results were expressed as ROI intensity per μm^2^ area and plotted in Prizm software. Significant differences in the fluorescence intensity of ROI were tested by Student’s t test.

### Quantitative real-time RT-PCR (qRT-PCR) for gene transcription

Total RNA was extracted from HELF (2 × 10^6^) at 0, 12 and 24 h after VZV inoculation using Trizol reagent (Invitrogen). The RNA samples were treated with TURBO™ DNA-free kit (Ambion) to remove genomic DNA according to the manufacturer’s instructions. The first strand of cDNA was synthesized from 2 μg of DNase treated RNA by random hexamer and M-MLV reverse transcriptase (Invitrogen). Primer sets used to amplify VZV genes were as follows. ORF4: 5′-CCACGGGAGAAAGAAATGAT-3′ and 5′-CGTACCGAGT CAATGGTCAC-3′; ORF23: 5′-ATAGGGACAGTCTCGGCAAA-3′ and 5′-GCTGATG CCAGAAGCATTTA-3′; ORF28: 5′-TCTGTGGCGCTCAATAACCTC-3′ and 5′-TACTT GCCGAGTGTTTACGC-3′; ORF51: 5′-TGTGTACATACGCGGGAGTT-3′ and 5′-CGTA CATCCTCGTGTTCAGG-3′; ORF61: 5′-TTCGGATAATACCTGCACCA-3′ and 5′-AGCAGAAGTCGTGCAAACAC-3′; ORF62: 5′-CAGACGATCATGTGGTTTCC’ and 5′-CGTCAAGTGGCATCGTTATT-3′; ORF68: 5′-CCCAAGGCCAAAGACTCA-3′ and 5′-TGCGTCTCCCGTACAGGTTA-3′. Primer sets for *Med25* are 5′-CAGCAGTCAGTCTCCAATAAG-3′ and 5′- TTCTCGCCATGATTCACG-3′; *Cdk8*: 5′-TTGGAGCAAGGCATTATACC-3′ and 5′-AGCATGTGGATTGGAACG-3′. Quantitative real-time reverse transcription PCR (qRT-PCR) amplification was performed using a Bio-Rad iCycler iQTM optical (one cycle of 94 °C for 5 min for the pre-denaturation step; 45 cycles of 94 °C for 30 s and 60 °C for 1 min) for VZV genes or using ABI 7900HT Real-Time PCR System (Applied Biosystems) (one cycle of 95 °C for 2 min for the pre-denaturation step; 40 cycles of 95 °C for 5 s and 60 °C for 30 s) for *med25* and *cdk8* genes at the First Core Research Laboratory, NTU. Relative expression of the qRT-PCR product was determined with the comparative 2^−ΔΔCt^ method after normalization to *Gapdh* mRNA (for VZV genes) or human *β*-*actin* mRNA (for *Med25* and *Cdk8* genes) expression.
